# Bacterial adhesion on fissure sealants: Effects of exposure to acidic drink

**DOI:** 10.4317/jced.54818

**Published:** 2018-06-01

**Authors:** Marco Colombo, Alberto Dagna, Domenico Molino, Claudio Poggio, Diego Maiolatesi, Giampiero Pietrocola

**Affiliations:** 1 Department of Clinical-Surgical, Diagnostic and Pediatric Sciences – Section of Dentistry, University of Pavia, Pavia, Italy; 2 Department of Molecular Medicine, Unit of Biochemistry, University of Pavia, Pavia, Italy

## Abstract

**Background:**

Adherence of bacteria to teeth surface is considered an important step in the development of caries and the use of fissure sealants is crucial for the prevention of caries in occlusal surfaces of molars and premolars. The aim of this study was to investigate and compare the adherence of *Streptococcus mutans* to different fissure sealants, after acidic drink exposure.

**Material and Methods:**

The tested materials were Fissurit, Fissurit FX, Grandio Seal, Fuji Triage, Constic. Bacterial suspension was deposited onto each material and the adhesion was evaluated trough the colony forming units (CFUs) determination with or without acidic drink exposure.

**Results:**

The tested materials showed different behaviors with significant differences. Bacterial adherence values of the untreated materials were very dissimilar: Fuji Triage and Constic materials showed the better results (*P*<0.05).

**Conclusions:**

Surface alteration after acidic drink exposure, changed the bacterial adhesion (except for Grandio Seal): Fissurit, Fissurit FX and Fuji Triage decreased their susceptibility to be colonized by *S. mutans* (*P*<0.05); on the contrary, Constic increased up to ~4 times the bacterial adhesiveness respected to the untreated control (*P*<0.05).

** Key words:**Acidic drinks, bacterial adhesion, fissure sealants, Streptococcus mutans.

## Introduction

Fissure sealants are widely used in dentistry for the prevention of caries in occlusal surfaces of molars and premolars and their effectiveness has been proved in many studies (1-4). They provide a physical barrier between the oral environment and deep pits or fissures of teeth, which are the main location of initial caries (5). *Streptococcus mutans* has been identified as the first responsible for the initiation of tooth decay, within the complex formation of a dental biofilm (6,7). The adhesion of bacteria to tooth surfaces or dental materials is considered the essential prerequisite for the formation of a cariopathogenic biofilm (8,9). Fissure sealants should reveal a low susceptibility to adhere to oral bacteria and, ideally, they should also exhibit antibacterial properties that may amplify their potential to prevent caries (10). The formation of dental plaque is a complex phenomenon involving microbial adhesion to oral surfaces; the plaque composition is an ensemble of salivary conditioning film and the initially adhering bacteria. If this former film is disrupted, the entire plaque and bacteria adhering on it may collapse and a clean surface results (11). After the initial adhesion, a firm anchorage between bacteria and oral surface has to be established by specific chemical interactions with complementary surface components; finally the colonization occurs to form a biofilm (12). The biofilm mode of growth protects the bacterial plaque against phagocytosis and antibiotics; the detachment of this film is the only way to destroy this bacterial environment and to prevent the formation of caries and periodontal disease (13,14).

Many different restorative materials are incorporated in the mouth: plaque formation has been investigated in conjunction with several properties of these materials such as surface roughness, electrical property, hydrophobicity, surface free-energy, and fluoride release (15-20). Dental materials restoration may influence the primary plaque formation because materials with various topography and surface chemistry may facilitate initial bacterial adhesion (21-23). Since 1960s, fissure sealants are considered a highly effective method for the prevention of dental caries (24). Today two main types of sealants are used: resin-based and glass ionomer cement (GIC) sealants (25). Fluoride-releasing sealants were introduced in the 1970s to increase the caries-preventing effect (26). Current commercial fluoride-releasing sealants contain either a soluble fluoride salt such as sodium fluoride (NaF) or fluoride-releasing glass filler or both. Various in vitro studies reported that these materials could release fluoride and inhibit demineralization of the adjacent tooth structure (27).

The aim of this study was to evaluate and compare adhesion of *Streptococcus mutans* on different fissure sealants after acidic drink exposure. The null hypothesis of the study was that there are no significant differences on bacterial adhesion values among the different fissure sealants after exposure to acidic drink.

## Material and Methods

-Specimens’ preparation

Five different fissure sealants were evaluated in this study ( Table 1). All materials were polymerized according to manufacturers’ instructions into silicon rings (height 2 mm; internal diameter 6 mm; external diameter 8 mm) to obtain specimens identical in size. Cavities of these rings were slightly overfilled with material, covered with Mylar strip (Henry Schein, Melville, NY, USA), pressed between glass plates and polymerized for 40 seconds on each side using a curing unit (D-Light Pro, GC Corp., Tokyo, Japan). One light polymerization mode was used for each material: 1400 mW/cm2 for 20 s. The intensity of the light was verified with a radiometer (SDS Kerr, Orange, CA, USA). The light was placed perpendicular to the specimen surface, at distance of 1 mm. Thirty specimens of each material were prepared in this manner. After polymerization and during the experimentation, the specimens were stored in distilled water at 37°C and 100% humidity. All specimens were sterilized with ethylene oxide gas/ethanol (70%) and packed in dry plastic sterile bags before being tested with bacteria. Each material was tested 4 weeks after polymerization. A polystyrene disc was used as control group (Thermanox plastic coverlips, Rochester, NY, USA).

**Table 1:**
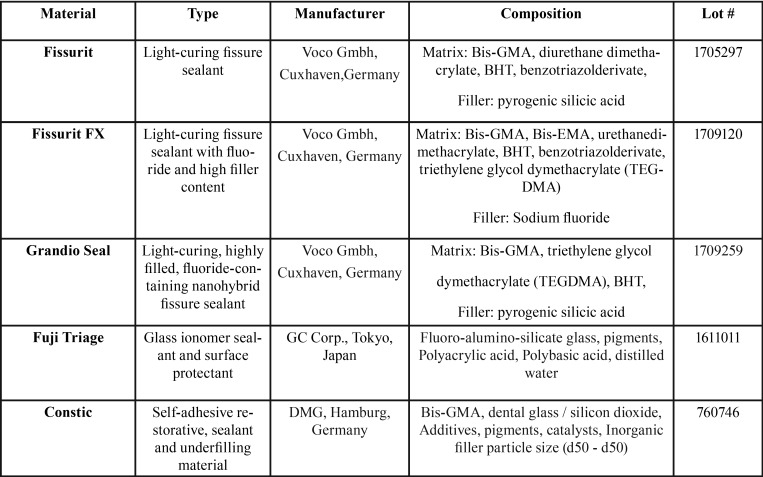
Fissure sealants tested in this study.

-Immersion in acidic drink

Specimens of each fissure sealant were divided into 3 subgroups (n=10): group 1 as control, group 2 was immersed in 50 ml of acidic drink (Coca Cola, The Coca Cola Company, Italy) for 1 day, group 2 was immersed in 50 ml of the same acidic drink for 7 days. All specimens were stored in distilled water for 5 days to reduce the potential antibacterial influence of residual cytotoxic acidic drink constituents.

-Bacterial growth condition

A strain of *Streptococcus mutans* (CCUG35176) was obtained from the Culture Collection of the University of Göteborg (CCUG) and used for the in vitro adhesion tests. *S. mutans* was cultured in Brain Heart Infusion (BHI, Difco, CA, USA) supplemented with 10% (v/v) heat-inactivated horse blood serum (Oxoid, Milan, Italy) to improve its growth. The culture of *S. mutans* was statically incubated for 16 hours at 37°C under aerobic conditions. The bacterial cells were harvested by centrifugation and washed three times with PBS. This bacterial suspension was reduced at a final density of 1*107 cells/mL as determined by comparing the OD600nm of the sample with a standard curve relating OD600nm to cell number.

-Assessment of bacterial adhesion 

Adherent *S. mutans* were quantified as described before in a previous study (28). Briefly, 1 mL of the bacterial suspension (107 bacteria/mL) was seeded onto each sample placed at the bottom of a 24-well plate (Celbio, Milan, Italy) and incubated in static conditions at 37°C for 4 hours. After incubation, non-adhering bacteria were removed by gently washing the samples with PBS, the adhering one were dispersed into 1 mL sterile Ringer solution (Oxoid, Milan, Italy) by vortex for 5 minutes. Serial dilutions of the bacterial cells suspensions were prepared and 0.1 mL of each dilution was deposited onto BHI agar (Bacto agar, Difco, CA, USA) plates. The plates were incubated for 24 to 48 hours at 37°C and the number of colonies counted. The results are expressed as Colony Forming Units (CFU) per mL.

-Statistical analysis

Bacterial adhesion data were subjected to Analysis of Variance (One-way ANOVA) followed by Bonferroni’s post hoc tests. Analyses were performed using Prism 4.0 (GraphPad). Two-tailed *P* values of 0.05 were considered statistically significant.

## Results

**Figure 1:**
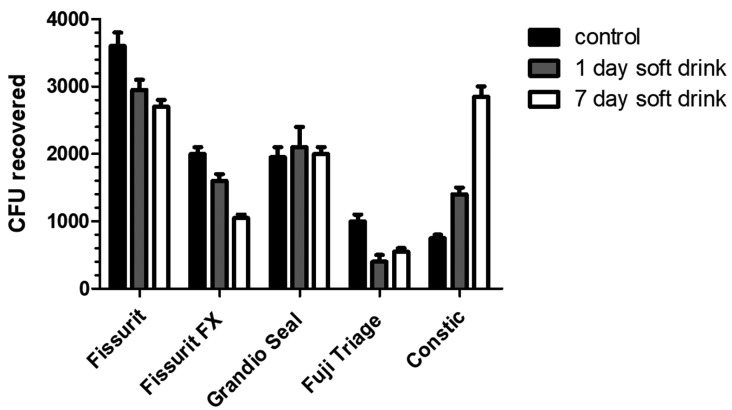
CFU values of *S. mutans* cells adherent to fissure sealants. Results were expressed as CFU/mL.

The ability of *S. mutans* strain to adhere to different sealant materials with or without soft drink treatment, are displayed in  Table 2 and in Fig. 1 as Colony Forming Units (CFU). The bacterial adherence values of the untreated materials (control group) were ranging from 7.5 x 102 to 3.6 x 103 CFU. Among these, Fuji Triage and Constic materials showed the lowest (*P*<0.05) bacterial adhesion values compared to the other analyzed materials. Instead, the highest (*P*< 0.05) bacterial adhesion values were calculated for Fissurit. Except for Grandio Seal, surface alteration by acidic drink changed the bacterial adhesion to the sealant materials tested. The treatment of Fissurit, Fissure FX and Fuji Triage with CoCa Cola for 1 or 7 days decreases in a time-dependent manner their susceptibility to be colonized by *S. mutans* (*P*<0.05). On the contrary, the treatment of Constic increased up to ~4 times the bacterial adhesiveness respected to the untreated control (*P*<0.05).

**Table 2:**
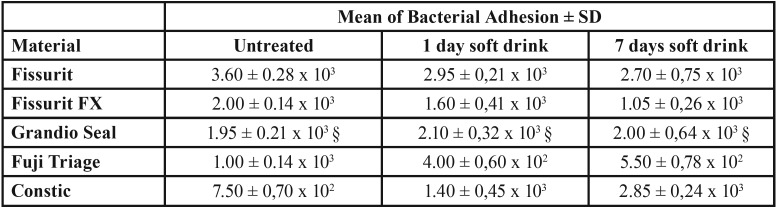
CFU values of *S. mutans* cells adherent to fissure sealants. Results were expressed as mean of bacterial adhesion ± SD. For each row, the symbol (§) denote no significant difference among data.

## Discussion

Bacterial plaque is a complex community of microorganisms organized as biofilm along tooth surface, embedded in an extracellular polymer matrix of host and bacterial origin: its establishment on hard surfaces is a process involving salivary pellicle formation, pellicle adsorption to the surface, passive transport of bacteria to the pellicle surface, co-adhesion and multiplication (29). The restorative materials used can influence the biofilm formation. It has been previously reported that polishing procedures applied on restorative materials can also influence bacterial adhesion by increasing the level of surface roughness and free-energy (30). Furthermore, there is some evidence showing how the superficial microstructure of the dental materials can influence bacterial adhesion and, consequently, biofilm formation (31). Fissure sealants are used to provide a physical barrier between the oral environment and deep pits or fissures of teeth, where bacteria can adhere and produce of initial caries (5). They should generally show a low susceptibility to adhere to oral bacteria and, moreover, they should also demonstrate an antibacterial effect that may amplify their potential to prevent caries (10). On the other side, it’s known that Coca-Cola soft drinks cause an increase of the surface roughness of the sealant materials (32). Coca Cola (pH 2.3) is widely consumed by the population and it’s got high erosive potential due to its low pH and fluoride/calcium concentrations (33). The oral cavity is for sure the suitable environment for evaluating the behavior of dental materials and it’s well known that, during consumption, beverages contact only with the tooth surface and restorative materials for a short time before they are washed away by saliva, but in vitro models are very important for providing insight into the fundamental mechanisms of biodegradation (32).

The present investigation showed different results between tested sealants ( Table 2, Fig. 1). The bacterial adherence values of the untreated materials (control group) were ranging from 7.5 x 102 to 3.6 x 103 CFU; in other terms, significant differences in bacterial adhesion values were found: Fuji Triage and Constic materials showed the lowest (*P*<0.05) bacterial adhesion values compared to the other analyzed material, while the highest (*P*< 0.05) bacterial adhesion values were calculated for Fissurit.

The phosphoric acid found in Coca Cola beverage could induce softening of the bisphenol-A-glycidyl methacrylate (Bis-GMA) polymers in resin sealants, which could result from the leaching of diluent agents, such as triethylene glycol dimethacrylate (32). Additionally, softening of the resin matrix could favor the displacement of inorganic fillers, contributing to the formation of a rough surface.

Surface alteration by Coca Cola acidic drink changed the bacterial adhesion to the sealant materials tested, except for Grandio Seal. The immersion in acidic drink of Fissurit, Fissurit FX and Fuji Triage for 1 or 7 days decreased in a time-dependent manner their susceptibility to be colonized by *S. mutans* (*P*<0.05). On the contrary, the immersion in acidic drink of Constic increased up to ~4 times the bacterial adhesiveness respected to the untreated control (*P*<0.05).

A previous study (34) showed a reduction in bacterial adhesion to glass ionomers after artificial aging/erosion and no statistically significant differences between baseline and after immersion in acidic drink were found for Grandio Seal (*P*>0.05). In this study (34) and in the study of Cavalcante Medeiro (32), glass ionomers have shown an increase in surface roughness after erosion/aging regimens. Furthermore, in some studies (34,28), no significant correlations are found between bacterial adhesion and roughness (Ra).

In previous studies it has been seen that Fuji Triage shows a higher fluoride release compared to other fissure sealants (35). The lower bacterial adhesion to Fuji Triage could be related to the greater amount of fluoride release.

The behaviour of fissure sealants is very different and no similarities were found at baseline or after exposure to acidic drinks like Coca Cola. Acidic exposure generates erosions or surface modifications of materials but in some cases, thanks to intrinsic properties of fissure sealants no increase of bacterial adhesion was found, so they could be considered in any case ideal materials for preventing caries of pits or fissures in occlusal surfaces of molars and premolars.

## Conclusions

Within the limitations of this study, all tested materials may be considered ideal for preventing caries in occlusal surfaces of molar and premolars, thanks to their antibacterial effect that induces a low adhesion of *S. mutans*, even if exposed to acidic substances that erode their surface.
